# Mesoscopic amorphous particles rather than oligomeric molecular aggregates are the cause of laser-induced crystal nucleation

**DOI:** 10.1073/pnas.2207173119

**Published:** 2022-07-25

**Authors:** Zhiyu Liao, Klaas Wynne

**Affiliations:** ^a^School of Chemistry, University of Glasgow, Glasgow G12 8QQ United Kingdom

Urquidi et al. (1) describe the use of a laser to induce nucleation through a process referred to as optical trapping induced crystallization, while simultaneously measuring Raman spectra of the solution, intermediates, and crystal. There are issues with the experimental methodology and the interpretation of the results that we would like to address.

**Fig. 1.**
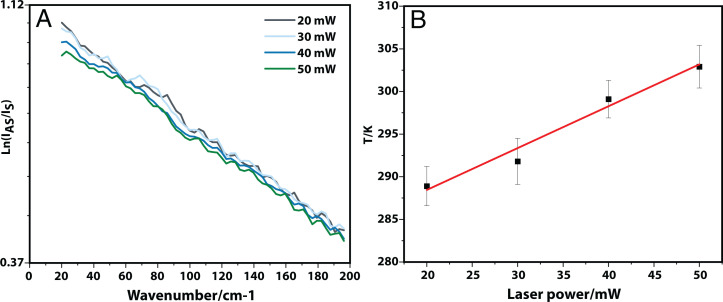
Temperature rise estimation using anti-Stokes and Stokes Raman scattering. (*A*) Intensity ratio of anti-Stokes and Stokes Raman scattering of a saturated glycine/D_2_O solution in the low-frequency region on a natural logarithmic scale. The sample has a thickness of 120 μm and is held between two coverslips. (*B*) Resultant temperature in the laser focus as a function of laser power.

Raman scattering is a weak process, and, in order to maximize time resolution and signal-to-noise, the authors employ a high-power laser (1 W/532 nm). It is stated that “the temperature of water at laser focus does not increase” (1). The absorption coefficient of water at 532 nm is 0.0447 m^−1^ (2), and using equation 20 of ref. 3 with a 100-μm sample on a 100-μm glass window predicts a temperature rise of 163 K and a characteristic heating spot radius of 77 μm. To support this back-of-the-envelope calculation, we carried out measurements of the temperature rise using Raman scattering (Fig. 1) in an aqueous glycine solution irradiated with a laser similar to that used by Urquidi et al. (1) and estimate a temperature rise of 400 K for a 1-W laser power, broadly consistent with the numerical estimation. Therefore, one can confidently conclude that irradiating a 100-μm sample of aqueous glycine with a 1-W 532-nm laser will lead to significant heating (up to and including boiling) and vigorous convection of the sample. This may well result in gradual evaporation despite the use of a coverslip enclosing the sample.

The authors correctly state that the optical gradient force is insufficient to trap single molecules (1). Therefore, it is proposed that the laser instead traps glycine–water aggregates. This would then give rise to an increase of the concentration, eventually resulting in the nucleation of a crystal. However, particles with a radius of <1 μm have insufficient trap depth to overcome Brownian motion (4), and hence it is impossible to optically trap nanometer-sized molecular clusters consisting of 1 to 20 glycine molecules. A possible exception would be laser-induced phase separation near a liquid–liquid critical point (3, 5), but there is no evidence for liquid–liquid phase separation in aqueous glycine solutions.

**Fig. 2.**
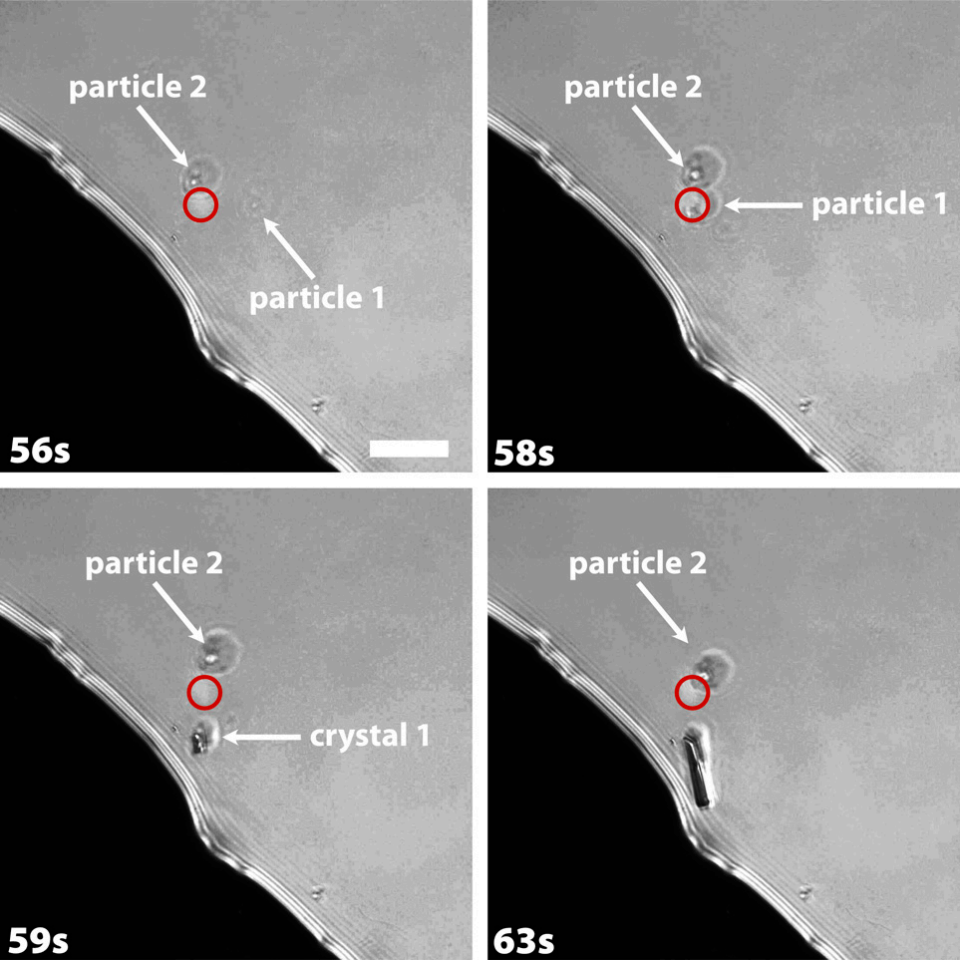
Laser-induced nucleation in an aqueous glycine solution. Micrometer-sized amorphous glycine particles form in saturated glycine/D_2_O solutions after high-power laser irradiation or aging (6). Here, two particles (labeled 1 and 2) diffuse into the laser focus (red circle) triggering nucleation, followed by crystal growth. The laser has a wavelength of 532 nm and a power of 50 mW. (Scale bar, 10 μm.)

Recently, it was shown that supersaturated aqueous glycine solutions form amorphous particles (6), which, when touched by a laser (50 mW and 532 nm used for simultaneous Raman spectroscopy), are optically trapped and nucleate a glycine crystal. Fig. 2 shows an example in which two amorphous particles are seen, and, as each is pulled into the laser focus, it nucleates a crystal.

In light of these considerations, it is likely the same phenomena are taking place in the work of Urquidi et al. (1). The reported randomness of the nucleation process is due to the random trapping of amorphous aggregates and not due to the inherent physical processes associated with nucleation. The conclusion that the laser optically traps molecular aggregates is not valid. However, the reported molecular dynamics simulations may well have a bearing on amorphous particles.
